# Human germ cell tumours: expression of **γ**-glutamyl transpeptidase and sensitivity to cisplatin

**DOI:** 10.1038/sj.bjc.6690653

**Published:** 1999-09

**Authors:** M H Hanigan, H F Frierson, V M Abeler, J Kaern, P T Taylor

**Affiliations:** Departments of Cell Biology, Obstetrics and Gynecology, and Pathology, University of Virginia, Charlottesville, 22908, USA; Departments of Pathology and Gynecologic Oncology, The Norwegian Radium Hospital, Oslo, Norway

**Keywords:** glutathione, human tumours, platinum-based therapy, chemotherapy

## Abstract

Previous studies have shown that the enzyme γ-glutamyl transpeptidase (GGT) is essential for the nephrotoxicity of cisplatin. This study was designed to determine whether GGT activity is necessary for the therapeutic effect of the drug. The relationship between GGT expression and clinical response to platinum-based chemotherapy was examined in 41 human germ cell tumours. Sections of formalin-fixed, paraffin-embedded tumours were immunohistochemically stained with an antibody directed against human GGT. There was no expression of GGT in any of the 17 seminomas or four dysgerminomas; whereas, 12/12 ovarian yolk sac tumours and 4/4 embryonal carcinomas of the testis were GGT-positive. In stage I tumours fewer tumour cells expressed GGT than in later stage tumours. In four germ cell tumours of mixed histology, the seminomatous and dysgerminoma areas were GGT-negative while the areas of the tumour with yolk sac or embryonal histology contained GGT-positive tumour cells. The patients with seminomas or dysgerminomas who were treated with cisplatin-based chemotherapy, all had a complete response despite the absence of GGT expression in these tumours. Fifteen of the 16 patients with yolk sac or embryonal carcinomas received cisplatin-based chemotherapy following surgery. Twelve had a complete response, while three failed to respond to platinum-based therapy. There was no correlation between the level of GGT-expression and response to therapy in this group. Three of the four patients with tumours of mixed histology were treated with cisplatin-based therapy, and had a complete response. Therefore, expression of GGT is not necessary for the therapeutic effect of cisplatin in germ cell tumours. The results from this study suggest that systemic inhibition of GGT would inhibit the nephrotoxic side-effect of cisplatin without interfering with its activity towards germ cell tumours. © 1999 Cancer Research Campaign


*British Journal of Cancer* (1999) **81**(1), 75–79
© 1999 Cancer Research Campaign
Article no. bjoc.1999.0653

### Human germ cell tumours: expression of γ-glutamyl transpeptidase and sensitivity to cisplatin

**MH Hanigan[1,2], HF Frierson Jr[3], VM Abeler[4], J Kaern[5] and PT Taylor Jr[2]**

Departments of [1]Cell Biology, [2]Obstetrics and Gynecology, and [3]Pathology, University of Virginia, Charlottesville, VA 22908, USA; Departments of [4]Pathology and [5]Gynecologic Oncology, The Norwegian Radium Hospital, Oslo, Norway

**Summary** Previous studies have shown that the enzyme γ-glutamyl transpeptidase (GGT) is essential for the nephrotoxicity of cisplatin. This study was designed to determine whether GGT activity is necessary for the therapeutic effect of the drug. The relationship between GGT expression and clinical response to platinum-based chemotherapy was examined in 41 human germ cell tumours. Sections of formalin-fixed, paraffin-embedded tumours were immunohistochemically stained with an antibody directed against human GGT. There was no expression of GGT in any of the 17 seminomas or four dysgerminomas; whereas, 12/12 ovarian yolk sac tumours and 4/4 embryonal carcinomas of the testis were GGT-positive. In stage I tumours fewer tumour cells expressed GGT than in later stage tumours. In four germ cell tumours of mixed histology, the seminomatous and dysgerminoma areas were GGT-negative while the areas of the tumour with yolk sac or embryonal histology contained GGT-positive tumour cells. The patients with seminomas or dysgerminomas who were treated with cisplatin-based chemotherapy, all had a complete response despite the absence of GGT expression in these tumours. Fifteen of the 16 patients with yolk sac or embryonal carcinomas received cisplatin-based chemotherapy following surgery. Twelve had a complete response, while three failed to respond to platinum-based therapy. There was no correlation between the level of GGT-expression and response to therapy in this group. Three of the four patients with tumours of mixed histology were treated with cisplatin-based therapy, and had a complete response. Therefore, expression of GGT is not necessary for the therapeutic effect of cisplatin in germ cell tumours. The results from this study suggest that systemic inhibition of GGT would inhibit the nephrotoxic side-effect of cisplatin without interfering with its activity towards germ cell tumours.

**Keywords:** glutathione; human tumours; platinum-based therapy; chemotherapy

Platinum-based combination chemotherapy has proven to be a highly effective modality in the treatment of both male and female germ cell tumours. Clinical use of cisplatin has increased the cure rate for patients with metastatic disease from 5% to 85% (Einhorn, 1990; Segelov et al, 1994). Unfortunately, cisplatin has both nephrotoxic and neurotoxic side-effects that limit its clinical utility. The nephrotoxicity requires that the patient have intravascular volume loading, active diuresis and renal function monitored during the course of therapy. The morbidity of this treatment would be significantly reduced if the nephrotoxicity of cisplatin could be inhibited without effecting the therapeutic efficacy.

The nephrotoxicity of cisplatin may involve a pathway that is distinct from anti-tumour activity of the drug. The therapeutic activity of cisplatin has been attributed to its ability to bind DNA, thereby killing dividing cells (Chu, 1994). The renal proximal tubule cells, which are the target of cisplatin toxicity, are a non-dividing cell population. The mechanism of cisplatin nephrotoxicity has not yet been elucidated. However, we have discovered that the nephrotoxicity of cisplatin can be blocked by inhibiting the enzyme γ-glutamyl transpeptidase (GGT), an enzyme present on the luminal surface of the renal proximal tubule cells (Hanigan et al, 1994*b*, 1996). GGT can be blocked in vivo with acivicin, a non-competitive inhibitor, or a high dose of glutathione can be used as a competitive inhibitor. Either treatment protects against cisplatin nephrotoxicity (Hanigan et al, 1994*b*). GGT is expressed on the surface of some ovarian tumours (Hanigan et al, 1994*a*). To determine whether GGT activity is an essential component of the therapeutic effect of cisplatin, we examined a series of human germ cell tumours for expression for GGT and analysed the relationship between GGT expression and response to platinum-based therapy. Germ cell tumours have historically been classified as seminomatous (testicular seminoma and ovarian dysgerminoma) and non-seminomatous (ovarian yolk sac tumour, embryonal carcinoma of the testis, choriocarcinoma and teratoma). In this study patients with seminomatous tumours were grouped together due to some histological and clinical similarities. Likewise the non-seminomatous testicular tumours (embryonal and mixed tumours) were grouped with ovarian yolk sac tumours and mixed tumours. Expression of GGT was determined immunohistochemically in formalin-fixed, paraffin-embedded tissues with the antibody GGT129. The GGT129 antibody was developed in our laboratory and is directed against a 20-amino acid peptide at the C-terminus of the heavy subunit of human GGT (Hanigan et al, 1996). We have shown that the level of immunohistochemical staining correlates with the level of GGT activity as measured biochemically (Hanigan et al, 1996). In this study, percentage of tumour cells staining positive was analysed in relation to tumour histology, the stage and grade of the tumour, and response to therapy.

*Received 3 September 1998*
*Revised 12 January 1999*
*Accepted 20 February 1999*

*Correspondence to:* MH Hanigan, Department of Cell Biology, School of Medicine Box 439, University of Virginia Health Sciences Center, Charlottesville, VA 22908, USA

### MATERIALS AND METHODS

### Human tissue

The Norwegian Radium Hospital (Oslo, Norway) provided sections of tumour tissue and clinical information for ten patients with yolk sac tumours. All other tissue specimens were obtained from archival files in the Department of Pathology at The University of Virginia Health Sciences Center (Charlottesville, VA, USA). Clinical information for patients treated at The University of Virginia Cancer Center were obtained from The McIntire Tumour Registry at the University of Virginia. Yolk sac tumours (also referred to as endodermal sinus tumours) and dysgerminomas were staged according to The International Federation of Gynecology and Obstetrics (FIGO) Stage Groupings for Primary Carcinomas of the Ovary (FIGO, 1988, 1989). Embryonal tumours and seminomas were staged according to the TNM staging system (American Joint Committee on Cancer, 1997). All tissues analysed were from the initial surgery and all were primary tumours, except one sample which was a lymph node metastasis from a male with a germ cell tumour of mixed histology. None of the patients had received any therapy prior to surgery.

### Immunohistochemistry

GGT129 is an affinity-purified polyclonal antibody directed against a 20-amino acid peptide corresponding to the C-terminus of the heavy subunit of GGT. The antibody was produced and purified in our laboratory (Hanigan et al, 1996). The procedure for immunohistochemical staining has been described previously (Hanigan et al, 1996). Briefly, 5-μm sections of formalin-fixed, paraffin-embedded tissue were deparaffinized and rehydrated. Endogenous peroxidase activity was inhibited with 0.3% hydrogen peroxide in methanol. Endogenous biotin was blocked with avidin and biotin blocking solutions (Avidin/Biotin Block Kit, Vector Laboratories Inc., Burlingame, CA, USA). Background staining was inhibited by incubating the sections for 10 min in phosphate-buffered saline containing 1.5% goat serum (Gibco, Grand Island, NY, USA). Affinity purified GGT129 antibody in 1% bovine serum albumin (BSA) was diluted 1:1000 relative to the starting serum concentration. The sections were incubated with the primary antibody for 45 min. Control slides were incubated in an equivalently diluted solution of 1% BSA. The primary antibody was localized with biotinylated anti-rabbit IgG and avidin-linked peroxidase (Vectastain Elite ABC Peroxidase Kit) and BioGenex Liquid DAB Substrate Kit (BioGenex, San Ramon, CA, USA). Slides were incubated for 3 min in 0.5% cupric sulphate to enhance the stain and counterstained in Gill's 3 Hematoxylin (Sigma, St Louis, MO, USA). All incubations were done at room temperature. A section of normal human kidney was included as a positive control with each set of sections stained.

The monoclonal mouse-antihuman antibody, CD68-KP1 (Dakopatts, Glostrup, Demark), was used to immunolocalize histiocytes in a subset of the dysgerminoma and seminoma tissues. The tissue sections were stained in the Ventana ES automated immunostainer (Ventana Medical Systems Inc., Tucson, AZ, USA). The CD68-KP1 antibody was diluted 1:100 and the tissues were incubated for 32 min at 42°C in the primary antibody.

### Histopathological assessment

The sections were analysed and scored for immunostaining by two observers (HFF and MHH). The percentage of tumour cells that were immunopositive was estimated for each sample.

### Response to treatment

Response to treatment was defined as complete response (CR) – no evidence of disease as determined by two observations not fewer than 4 weeks apart; stable disease (SD) – measurable lesions remain of stable size or do not increase more than 25% or decrease more than 50%; progressive disease (PD) – an increase of 25% or more in the size of one or more measurable lesions or the appearance of new lesions (Miller et al, 1981).

### Statistical analysis

The correlation between GGT expression and the histological classification of the tumours was analysed for significance with the $\chi^2$ test with Yates' correction for $2 \times 2$ contingency tables (Glantz, 1992). The correlation between the percentage of tumour cells that were GGT-positive and tumour stage (stage I vs stages II–IV) was analysed with a *t*-test. The correlation between GGT expression and the response of the tumours to chemotherapy was analysed for significance with both the Mann–Whitney rank sum test including a Yates correction for continuity and a correction for ties and by grouping tumours with low versus high levels of GGT expression and analysing the data with the Fisher exact test (Glantz, 1992).

### RESULTS

### Dysgerminomas and seminomas

Seventeen testicular seminomas and four ovarian dysgerminomas were evaluated for GGT expression and response to therapy. All of the tumour cells in these 21 malignancies were negative for GGT staining. GGT-positive non-neoplastic cells were observed in the stroma of many of these tumours. Immunohistochemical staining with CD 68 antibody revealed that the GGT-positive cells were histiocytes.

The patients with seminomas ranged in age from 25 to 57 years. All were treated surgically. Four patients with localized disease received no treatment following surgery. One of these four patients had a distant recurrence at 14 months and was treated with cisplatin–etoposide and bleomycin. He had a complete response with no evidence of disease at 5 years. Eleven patients were treated with radiation following surgery. Nine had no recurrence of disease. Two had distant metastasis identified 14–22 months after diagnosis and were then treated with cisplatin–etoposide–bleomycin. Both had a complete response. One patient had metastatic disease at the time of surgery. He was treated with cisplatin–etoposide–bleomycin. He had a complete response with no evidence of disease at 9 years. One patient received treatment elsewhere and was lost to follow-up.

Therefore, four patients with metastatic seminomas were treated with cisplatin-based chemotherapy. All four had a complete response despite the absent of GGT expression in these tumours. This high complete response rate to cisplatin-based chemotherapy among metastatic seminomas is similar to the response rates seen at other institutions (Einhorn, 1990; Segelov et al, 1994).

 © 1999 Cancer Research Campaign

**Table 1** Yolk sac tumours and embryonal carcinomas: GGT immunohistochemical staining and response to treatment

| Patient number | Age | Stage | GGT-positive | TMT | Response | Survival (months) | Status |
|---|---|---|---|---|---|---|---|
| Yolk sac tumour of the ovary | | | | | | | |
| 1 | 21 | Ia | 5% | PVB | CR | 82 | NED |
| 2 | 12 | Ia | 25% | PE | CR | 24 | NED |
| 3 | 17 | Ic | 25% | CAOS | CR | 140 | NED |
| 4 | 19 | Ic | 70% | PE | CR | 72 | NED |
| 5 | 11 | Ic | 40% | PE | CR | 48 | NED |
| 6 | 15 | Ic | 10% | PE | CR | 20 | NED |
| 7 | 24 | IIc | 75% | PEB+RAD | CR | 96 | NED |
| 8 | 69 | IIc | 50% | PE | SD | 13 | DOD |
| 9 | 40 | IIIc | 75% | PEB | CR | 35 | NED |
| 10 | 21 | IIIc | 75% | PE | SD | 13 | DOD |
| 11 | 32 | IV | 50% | PEB | CR | 83 | NED |
| 12 | 22 | IV | 90% | PVAC | PD | 6 | DOD |
| Embryonal carcinoma of the testis | | | | | | | |
| 13 | 20 | II | 5% | PEB/PNB | CR | 83 | NED |
| 14 | 24 | II | 25% | PNB | CR | 149 | NED |
| 15 | 27 | III | 90% | PEB | CR | 97 | NED |
| 16 | 20 | III | 100% | PEB | CR | 21 | NED |

TMT, treatment; P, cisplatin; E, etoposide (VP16); B, bleomycin; V, vincristine; A, actinomycin D; C, cyclophosphamide; O, oncovin; S, adriamycin; RAD, radiation therapy; N, vinblastine; CR, complete response; SD, stable disease; PD, progressive disease; NED, no evidence of disease; DOD, dead of disease.

The four patients with dysgerminomas ranged in age from 14 to 74 years. Two were diagnosed at stage Ia and two were stage IIIc. All four patients were treated with radiation following surgery. Three had no subsequent evidence of disease. One of the patients diagnosed at stage Ia was found to have distant metastasis 6 months after surgery. She was diagnosed in 1978 prior to the widespread availability of cisplatin. She died 1 year after diagnosis.

### Ovarian yolk sac tumours and embryonal carcinoma of the testis

Twelve ovarian yolk sac tumours and four embryonal carcinomas of the testis were evaluated for expression of GGT (Table 1). Patients ranged in age from 11 to 69 years of age. Immunohistochemical staining for GGT showed that all of the tumours contained a mixture of GGT-positive and GGT-negative tumour cells. This finding is in contrast to normal female germ cells and sperm that are GGT-negative (Hanigan et al, 1996). The stage I ovarian yolk sac tumours contained a significantly lower percentage of GGT-positive cells than the stage II, III and IV tumours ($P < 0.01$, Figure 1). The testicular embryonal carcinomas occurred in patients from 20 to 27 years of age. Among the embryonal carcinomas, tumours diagnosed at stage II contained only 5–25% GGT-positive cells, while the two neoplasms diagnosed at stage III contained 90–100% GGT-positive cells (Table 1, Figure 2).

All patients, except one, diagnosed with yolk sac tumour or embryonal carcinoma were treated with cisplatin-based combination chemotherapy. The exception was patient no. 3 (Table 1) who was treated with combination chemotherapy that did not include cisplatin. Of the 15 patients treated with cisplatin, 12 have had a complete response to treatment with no subsequent evidence of disease for 20 months or longer. One patient died 6 months following the diagnosis and two died at 13 months. There is no statistically significant correlation between GGT expression and response to cisplatin-based chemotherapy.

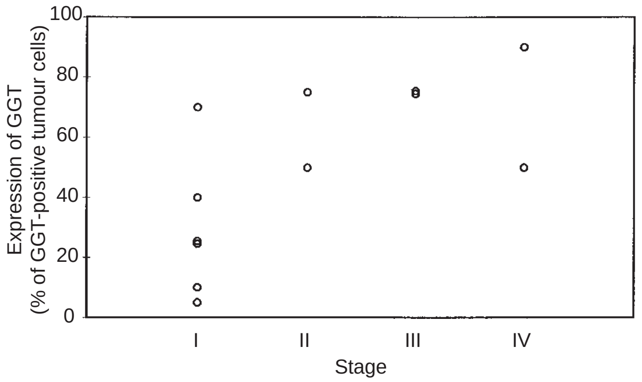

**Figure 1** Expression of GGT versus tumour stage in ovarian yolk sac tumours. The percentage of tumour cells that were GGT-positive is shown for stage I–IV yolk sac tumours. Expression of GGT was significantly lower in stage I tumours in comparison to higher stage tumours ($P < 0.01$)

### Tumours with mixed histology

Germ cell tumours with mixed histology were also examined for GGT (Table 2). One tumour, from a 16-year-old female (patient no. 17), contained both yolk sac tumour and dysgerminoma. Within the yolk sac portion of the tumour, 5% of the tumour cells were GGT-positive. All of the dysgerminoma cells were GGT-negative. Two males, ages 21 and 29, had mixed embryonal carcinoma and seminoma. The embryonal portions of the tumours were 90% and 50% GGT-positive while the seminomatous portions were negative. A third male, age 22 years, had a mixed germ cell tumour with embryonal, choriocarcinomatous and teratomatous elements. The tumour tissue that was analysed was from a lymph node metastasis consisted entirely of embryonal carcinoma. Ten per cent of the tumour cells in the lymph node were GGT-positive.

Patient no. 18 (Table 2) was diagnosed with stage I disease and did not receive further treatment. The three patients with advanced

© 1999 Cancer Research Campaign 

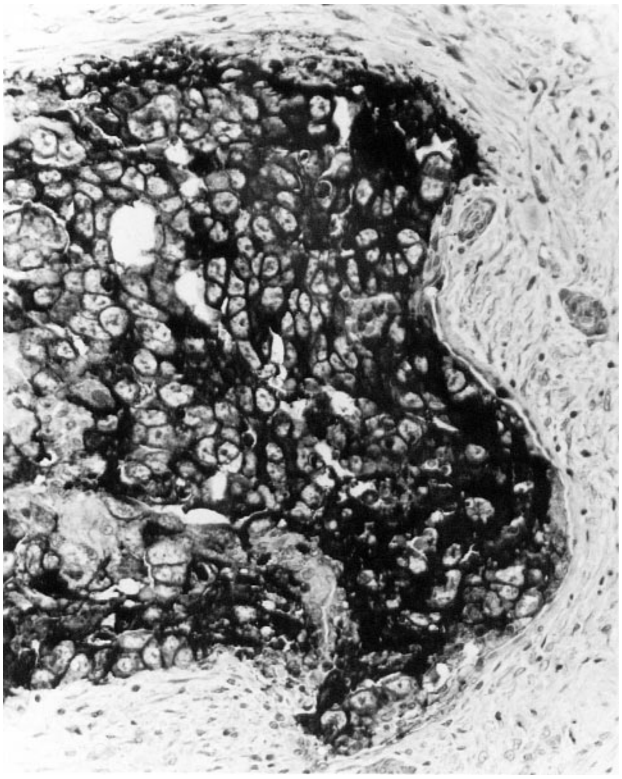

**Figure 2** Immunohistochemical detection of GGT in an embryonal carcinoma. The embryonal carcinoma from patient no. 16. Note that in many of the tumour cells the stain is localized to the surface membrane which is the location of GGT in normal tissues in which it is expressed disease were all treated with cisplatin–etoposide and bleomycin. All had a complete response with no evidence of disease more than 5 years after diagnosis.

### GGT expression and response to cisplatin-based chemotherapy

As shown in Table 3 there is an exact correlation between the histology of the tumour and the presence of GGT-positive tumour cells ($P < 0.001$). All of the tumour cells in the seminomas or dysgerminomas were GGT-negative, while each of the yolk sac tumours and embryonal carcinomas contained GGT-positive cells. Among the tumours of mixed histology the seminomas and dysgerminomatous components were GGT-negative, while the yolk sac and embryonal components contained GGT-positive tumour cells.

None of the patients in this study had treatment prior to surgery. Of the 22 patients in this study who received cisplatin-based therapy following surgery, 19 (86%) had no further evidence of disease. The three patients who had stable or progressive disease had been diagnosed with yolk sac tumours of the ovary. There is no correlation in that group between the percentage of tumour cells that were GGT-positive and response to therapy. Patients with seminomas, which are completely GGT-negative, and patients with embryonal carcinomas, which are GGT-positive, all responded to cisplatin-based combination chemotherapy. Based on these data we conclude that expression of GGT in the tumour cells is not necessary for the tumour to respond to cisplatin-based chemotherapy.

### DISCUSSION

In this study human germ cell tumours were analysed for GGT expression and response to therapy to determine whether expression of GGT is necessary for response to cisplatin-based chemotherapy. Immunohistochemical staining showed that all of the seminomas and dysgerminomas were GGT-negative. The yolk sac and embryonal germ cell tumours contained GGT-positive tumour cells. In germ cell tumours of mixed histology the seminomatous and dysgerminomatous components were GGT-negative while the yolk sac and embryonal components contained GGT-positive tumour cells. Twenty-two of the patients in this study were treated with cisplatin-based chemotherapy. Nineteen have had no further evidence of disease for 20 months or more. Three patients with yolk sac tumours failed to achieve a complete response. Patients with seminomas, which are GGT-negative, and patients with mixed tumors containing seminomatous and dysgerminomatous components all had a complete response to cisplatin-based chemotherapy. Therefore, expression of GGT is not necessary for germ cell tumours to respond to cisplatin-based chemotherapy.

Our previous studies have shown that sperm and normal female oocytes are GGT-negative (Hanigan et al, 1996). Sepulveda and co-workers reported that GGT is expressed in midgestational yolk sacs of mouse embryos and in mouse yolk sac carcinoma cells (Sepulveda et al, 1995). All of the yolk sac and embryonal tumours in this study contained GGT-positive cells. Van'T Sant

**Table 2** Germ cell tumours with mixed histology: GGT immunohistochemical staining and response to treatment

| Patient number | Age | Stage | Histology | GGT-positive | TMT | Response | Survival (months) | Status |
|---|---|---|---|---|---|---|---|---|
| Female | | | | | | | | |
| 17 | 16 | IIIc | Yolk sac | 5% | PEB | CR | 76 | NED |
| | | | Dysgerminoma | 0% | | | | |
| Male | | | | | | | | |
| 18 | 21 | I | Embryonal | 90% | None | CR | 97 | NED |
| | | | Seminoma | 0% | | | | |
| 19 | 29 | II | Embryonal | 50% | PEB | CR | 67 | NED |
| | | | Seminoma | 0% | | | | |
| 20[a] | 22 | II | Embryonal | 10% | PEB | CR | 80 | NED |

TMT, treatment; P, cisplatin; E, etoposide (VP16); B, bleomycin; CR, complete response; NED, no evidence of disease. [a]The pathology report for this patient stated that tumour was a mixture of embryonal carcinoma, teratoma and choriocarcinoma. The tissue examined for this study was a lymph node metastasis that included only embryonal carcinoma.

© 1999 Cancer Research Campaign

**Table 3** Human germ cell tumours: expression of GGT and response to cisplatin-based chemotherapy

| Histology | GGT-positive tumours/ total number of tumours analysed | Number of tumours treated with cisplatin-based chemotherapy | Complete response following cisplatin-based chemotherapy |
|---|---|---|---|
| Seminoma | 0/17 | 4 | 100% |
| Dysgerminomas | 0/4 | 0 | – |
| Yolk sac | 12/12 | 11 | 73% |
| Embryonal | 4/4 | 4 | 100% |
| Mixed histology | 4/4 | 3 | 100% |

and co-workers reported that GGT activity was elevated in serum from patients with non-seminomatous germ cell tumours (Van'T Sant et al, 1984). None of the patients in the study had liver metastasis prompting the investigators to suggest that the enzyme found in the serum was synthesized by the tumour. They found that stage III patients who had an elevated serum level of GGT at the time of diagnosis had significantly higher mortality than those without initially elevated GGT.

Tumours that are GGT-positive are able to use extracellular glutathione as a secondary source of cysteine (Hochwald et al, 1996). GGT cleaves the γ-glutamyl bond of extracellular glutathione, initiating its hydrolysis into its three constituent amino acids: glutamic acid, cysteine and glycine (Hanigan et al, 1993). The additional cysteine can be used to increase intracellular glutathione concentrations (Hanigan, 1995). There are conflicting data from in vitro studies regarding the relationship between GGT expression and cisplatin sensitivity (Godwin et al, 1992; Bailey et al, 1994; Tew et al, 1996). In vitro the effect of GGT is often masked by the extremely high concentration of cysteine in many tissue culture media. A recent clinical study of GGT expression in ovarian surface epithelial carcinomas showed that GGT expression in the tumour did not alter the response to primary cisplatin-based chemotherapy (Hanigan et al, 1998).

Inhibition of GGT blocks the nephrotoxicity of cisplatin (Hanigan et al, 1994*b*). The kidney contains several unique metabolic pathways which may be responsible for the nephrotoxicity of cisplatin. One such pathway is the mercapturic acid pathway (Dekant et al, 1995). Cleavage of glutathione-conjugated compounds by GGT is the first step in this pathway. Ongoing research in our laboratory is aimed at identifying the mechanism by which cisplatin is activated to a nephrotoxin.

In summary, GGT activity is essential for the nephrotoxicity of cisplatin but does not affect the response of germ cell tumours to cisplatin-based chemotherapy. If the nephrotoxicity of cisplatin is via a metabolic pathway that is specific to the kidney then it may be possible to block the nephrotoxicity of cisplatin without inhibiting its anti-tumour activity. Understanding the mechanism of cisplatin toxicity towards both germ cell tumours and non-dividing tissues such as the kidney will provide important information with which to further increase the therapeutic efficacy of this widely used anti-tumour agent.

### ACKNOWLEDGEMENTS

This work was supported by grant CA 57530 from the National Cancer Institute, NIH. We gratefully acknowledge the technical assistance of Dr Betty Gallagher for the immunohistochemical staining and for assistance with the statistical analysis.

### REFERENCES

American Joint Committee on Cancer (1997) *AJCC Cancer Staging Manual*, 5th Ed. Lippincott-Raven: Philadelphia

Bailey HH, Gipp JJ and Mulcahy RT (1994) Increased expression of gamma-glutamyl transpeptidase in transfected tumor cells and its relationship to drug sensitivity. *Cancer Lett* **87**: 163–170

Chu G (1994) Cellular responses to cisplatin. *J Biol Chem* **269**: 787–790

Dekant W, Vamvakas S and Anders MW (1995) Formation and fate of nephrotoxic and cytotoxic glutathione S-conjugates: cysteine conjugate beta-lyase pathway. In: *Advances in Pharmacology*, Vol. 27. Anders MW and Dekant W (eds), pp. 115–162. Academic Press: New York

Einhorn LH (1990) Treatment of testicular cancer: a new and improved model. *J Clin Oncol* **8**: 1777–1781

FIGO (1988) *Annual Report of the Results of Treatment in Gynecological Cancer*, Vol. 20. International Federation of Gynecology and Obstetrics: Stockholm

FIGO (1989) Annual report on the results of treatment in gynecological cancer. *Int J Gynecol Obstet* **28**: 189–193

Glantz SA (1992) *Primer of Bio-Statics*, 3rd edn. McGraw-Hill: New York

Godwin AK, Meister A, O'Dwyer PJ, Huang CS, Hamilton TC and Anderson ME (1992) High resistance to cisplatin in human ovarian cancer cell lines is associated with marked increase of glutathione synthesis. *Proc Natl Acad Sci USA* **89**: 3070–3074

Hanigan MH and Ricketts WA (1993) Extracellular glutathione is a source of cysteine for cells that express gamma-glutamyl transpeptidase. *Biochemistry* **32**: 6302–6306

Hanigan MH, Frierson HF Jr, Brown JE, Lovell MA and Taylor PT (1994a) Human ovarian tumors express gamma-glutamyl transpeptidase. *Cancer Res* **54**: 286–290

Hanigan MH, Gallagher BC, Taylor PT Jr and Large MK (1994b) Inhibition of gamma-glutamyl transpeptidase activity by acivicin in vivo protects the kidney from cisplatin-induced toxicity. *Cancer Res* **54**: 5925–5929

Hanigan MH (1995) Expression of gamma-glutamyl transpeptidase provides tumor cells with a selective growth advantage at physiologic concentrations of cyst(e)ine. *Carcinogenesis* **16**: 181–185

Hanigan MH and Frierson HF Jr (1996) Immunohistochemical detection of γ-glutamyl transpeptidase in normal human tissue. *J Histochem Cytochem* **44**: 1101–1108

Hanigan MH, Frierson HF Jr and Taylor PT Jr (1998) Expression of gamma-glutamyl transpeptidase in stage III and IV ovarian surface epithelial carcinomas does not alter response to primary cisplatin-based chemotherapy. *Am J Obstet Gynecol* **179**: 363–367

Hochwald SN, Harrison LE, Rose DM, Anderson M and Burt ME (1996) Gamma-glutamyl transpeptidase mediation of tumor glutathione utilization in vivo. *J Natl Cancer Inst* **88**: 193–197

Miller AB, Hoogstraten B, Staquet M and Winkler A (1981) Reporting results of cancer treatment. *Cancer* **47**: 207–214

Segelov E, Campbell J, Ng M, Tattersall M, Rome R, Free K, Hacker N and Friedlander ML (1994) Cisplatin-based chemotherapy for ovarian germ cell malignancies: the Australian experience. *J Clin Oncol* **12**: 378–384

Sepulveda AR, Habib GM, Damjanov A, Matacic S, Damjanov I, Lebovitz RM and Lieberman MW (1995) Expression of gamma-glutamyl transpeptidase in midgestational mouse yolk sac and mouse visceral yolk sac carcinoma cells. *Exp Cell Res* **219**: 494–498

Tew KD, Monks A, Barone L, Rosser D, Akerman G, Montali JA, Wheatley JB and Schmidt DE Jr (1996) Glutathione-associated enzymes in the human cell lines of the National Cancer Institute Drug Screening Program. *Mol Pharmacol* **50**: 149–159

Van'T Sant P, Sleijfer DT, Schraffordt Koops H, Suurmeijer AJH, Willemse PHB, De Bruijn HWA, Marrink J and Ockhiuzen T (1984) The pattern of γ-glutamyl transpeptidase, alkaline phosphatase, serum glutamyl oxalate transaminase and serum glutamyl pyruvate transaminase inpatients with disseminated non-seminomatous testicular tumors. *Eur J Cancer Clin Oncol* **20**: 209–215

© 1999 Cancer Research Campaign 
